# Three New *Ent*-Kaurane Diterpenes with Antibacterial Activity from *Sigesbeckia orientalis*

**DOI:** 10.3390/molecules29194631

**Published:** 2024-09-29

**Authors:** Zhong-Shun Zhou, Zhao-Jie Wang, Bei Tian, Yan-Yan Zhu, Mei-Zhen Wei, Yun-Li Zhao, Xiao-Dong Luo

**Affiliations:** 1 Yunnan Characteristic Plant Extraction Laboratory, Key Laboratory of Medicinal Chemistry for Natural Resource, Ministry of Education and Yunnan Province, School of Chemical Science and Technology, Yunnan University, Kunming 650091, China; zhouzhongshun195@outlook.com (Z.-S.Z.); wangzhaojie10111@163.com (Z.-J.W.); 15050597099@163.com (B.T.); 22021016018@mail.ynu.edu (Y.-Y.Z.); 22021016017@mail.ynu.edu.cn (M.-Z.W.); zhaoyunli@mail.kib.ac.cn (Y.-L.Z.); 2 State Key Laboratory of Phytochemistry and Plant Resources in West China, Kunming Institute of Botany, Chinese Academy of Sciences, Kunming 650201, China

**Keywords:** *Sigesbeckia orientalis*, *ent*-kaurane diterpenes, anti-MRSA, anti-VRE, synergic bioactivity

## Abstract

Three novel *ent*-kaurane diterpenes, namely sigesbeckin A–C (**1**–**3**), in conjunction with eight previously identified analogues (**4**–**11**), were isolated from *Sigesbeckia orientalis*. Their chemical structures were resolved through multiple spectroscopic analyses. All compounds were assessed for antimicrobial bioactivity against methicillin-resistant *Staphylococcus aureus* (MRSA) and vancomycin-resistant *enterococci* (VRE) strains. In particular, compounds **1** and **5** demonstrated moderate efficacy, with MIC values of 64 μg/mL. Moreover, compounds **3**, **5**, and **11** were found to synergize with doxorubicin hydrochloride (DOX) and vancomycin (VAN) against MRSA and VRE. The aforementioned findings offer valuable insights for the development of novel alternatives to antibiotics, which can effectively tackle the escalating issue of antibiotic resistance.

## 1. Introduction

The prevalence of MRSA and VRE, termed “superbacterial”, has escalated globally, expanding from tertiary care and university hospitals to smaller healthcare facilities over the past decades [1,2]. These pathogens are primary causative agents of a range of severe infections, including bacteremia, endocarditis, cutaneous and subcutaneous infections, osteoarticular infections, and nosocomial infections [3,4]. The escalating threat posed by these bacteria is compounded by their resistance to conventional antibiotics; MRSA exhibits resistance to *β*-lactam antibiotics, such as methicillin, while VRE shows resistance to glycopeptides, like vancomycin. This resistance severely limits the effectiveness of current treatments and complicates clinical management strategies [5,6]. Furthermore, MRSA and VRE have developed the ability to form biofilms, which serve as protective barriers against both the host immune system and the bactericidal effects of antibiotics. This adaptive strategy underscores the urgent need for innovative therapeutic approaches that not only target these bacteria effectively but also disrupt their biofilm formation to prevent resistance development [7,8,9]. The impact of these infections is significant: annually, MRSA infections exceed 80,000 cases, with over 11,000 fatalities, while VRE accounts for approximately 20,000 infections and 1300 deaths per year. Both MRSA and VRE have been identified by the CDC as critical threats requiring immediate attention [10]. In this context, the exploration of antimicrobial agents derived from natural sources offers a promising avenue for addressing the challenges posed by multi-drug-resistant pathogens [11]. Natural products have a long history of providing effective treatments for various diseases and have the potential to yield new compounds with unique mechanisms of action that can overcome antibiotic resistance [12]. The isolation and characterization of novel compounds from plants, microorganisms, and other natural sources could lead to the development of new therapeutic options that are effective against MRSA and VRE while minimizing the risk of inducing further resistance. 

The exploration of natural products for bioactive compounds has long been a rich field in the quest for novel therapeutic agents, offering promising leads for drug development [13]. From 1981 to 2019, the U.S. Food and Drug Administration (FDA) approved a total of 1394 small molecules for marketing, nearly half (46.7%) of which were derived from natural products or their derivatives [14]. This substantial contribution underscores the potential of nature as a source of innovative medicines. Various mechanisms may contribute to the antimicrobial activity exhibited by these natural products, ranging from disrupting cell membrane integrity to inhibiting essential enzyme functions [15,16,17]. 

Diterpenes, a class of secondary metabolites prevalent among natural products, are known for their diverse and significant pharmacological effects [18]. The genus *Sigesbeckia*, with four species, is particularly noteworthy; however, only three species—*S. orientalis* L., *S. pubescens* Makino, and *S. glabrescens* Makino—are found in China [19,20]. Phytochemical studies on these *Siegesbeckia* species have revealed them to be rich repositories of diterpenoid and sesquiterpenoid compounds [21,22,23,24]. Among these, *S. orientalis* stands out for its traditional use in Chinese medicine to treat gold sore (tetanus infection) and various inflammatory conditions [23,25,26,27,28].

**Figure 1:**
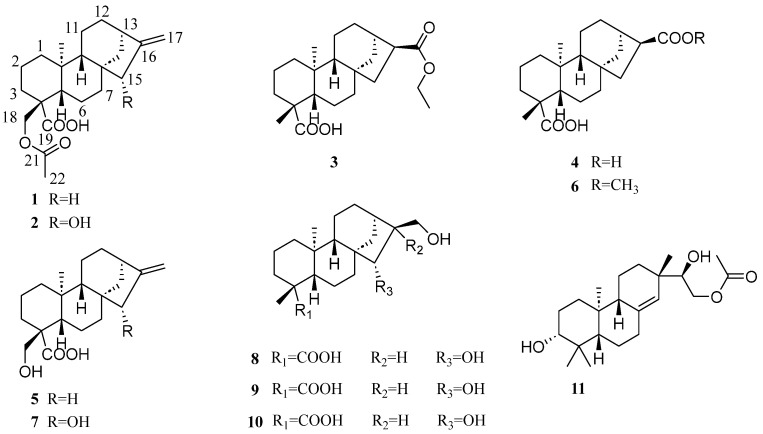
Compounds **1**–**11**.

In antimicrobial screening, *S. orientalis* demonstrated notable activity against MRSA. This prompted the isolation of three new diterpenes, named sigesbeckin A–C (**1**–**3**), characterized by a kaurene-type skeleton with varying substituents, alongside eight known analogs (**4**–**11**) from the plant (Figure 1). Compounds **1** and **5** showed inhibitory activity against both MRSA and VRE, with MIC values at 64 μg/mL. Remarkably, the results of antibacterial synergy experiments indicated that compounds **3**, **5**, and **11** exhibited enhanced activity when combined with DOX and VAN. This study provides a comprehensive description of the isolation, structural elucidation, and inhibitory effects of the compounds derived from *S. orientalis* against MRSA and VRE. The findings highlight the potential of *S. orientalis* as a source of effective antimicrobial agents, particularly in the context of rising antibiotic resistance. The discovery of sigebeckins A–C and their analogs not only broadens our understanding of the chemical diversity within this plant genus but also suggests new avenues for developing combination therapies to combat resistant bacterial strains. These insights are crucial as we seek to overcome the challenges posed by multidrug-resistant pathogens and underscore the enduring value of natural products in modern medicine.

## 2. Results and Discussion

The molecular formula of sigesbeckin A (**1**) was deduced to be C_22_H_32_O_4_ based on the observation of its ion peak at *m*/*z* 483.21942 [M + Na]^+^ (calculated for 383.21928) from its HRESIMS spectrum, which indicated the presence of seven degrees of unsaturation. Its IR absorption implied the presence of -CH_3_ (2971 cm^−1^), carbonyl (1746 cm^−1^), and double-bond (1655 cm^−1^) functional groups. Its ^1^H NMR spectrum (Table 1) displayed two methyl signals [*δ*_H_ 0.99 (H-20) and 2.06 (H-22)] and a double-bond signal at *δ*_H_ 4.79 (H-17). In its ^13^C NMR and HSQC spectrum, 22 carbon signals were observed and assigned to 2 carbonyl groups [*δ*_C_ 181.8 (C-19) and 171.0 (C-21)], two methyl carbons [*δ*_C_ 15.5 (C-20) and 20.8 (C-22)] and a terminal double bond carbon [*δ*_C_ 155.5 (C-16) and 103.2 (C-17)]. The aforementioned findings indicated the presence of three degrees of unsaturation, while the remaining four degrees of unsaturation implied a characteristic kaurane diterpene framework in compound **1**, resembling the known compound of 18-hydroxy-kauran-16-*ent*-19-oic acid (**5**) [29], except for the additional carbonyl and methyl signals. 

**Figure 2:**
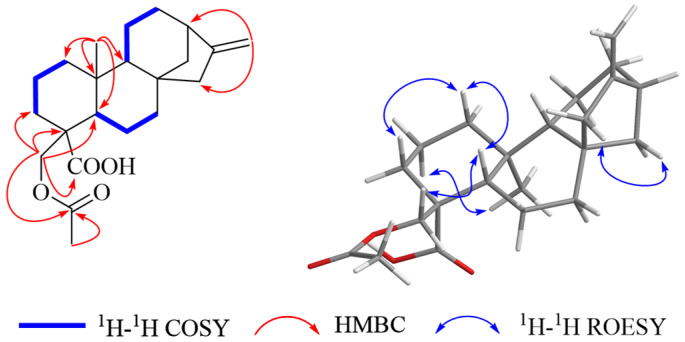
Key HMBC, ^1^H-^1^H COSY, and ^1^H-^1^H ROESY correlations of compound **1**.

The correlations of *δ*_H_ 2.06 (H-22) with *δ*_C_ 171.0 (C-21) in its HMBC spectrum (Figure 2) indicated the presence of an acetyl group. Moreover, the HMBC correlations of *δ*_H_ 4.01 (H-18a) and 3.49 (H-18b) with *δ*_C_ 171.0 (C-21), 181.8 (C-3′′) 32.3 (C-3), 47.5 (C-4), and 52.3 (C-5) implied the presence of an *O*-acetyl group in the C-18 position to meet the molecular formula of **1**. Then, the plane structure of compound **1** was established as 18-*O*-acetyl -kauran-16-*ent*-19-oic acid. The ROSEY spectrum of compound **1** (Figure 2) shows NOE correlations at *δ*_H_ 4.01 (H-18a) and 3.49 (H-18a) with 1.28 (H-5) and 1.10 (H-9), indicating the *α*-orientation of the -CH_2_OAc group at C-5, consistent with 18-hydroxy-kauran-16-*ent*-19-oic acid (**5**) [28]. The absolute configurations of compound **1** were determined as 4*R*, 5*S*, 8*S*, 9*R*, 10*S*, and 13*R* by comparing its optical rotation value with that of the known compound [30]. Then, diterpene **1** was deduced as shown and named sigesbeckin A.

**Figure 3:**
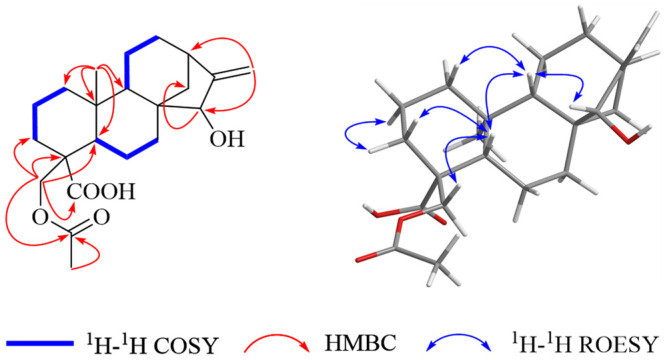
Key HMBC, ^1^H-^1^H COSY, and ^1^H-^1^H ROESY correlations of compound **2**.

Compound **2** possessed a molecular formula of C_22_H_32_O_5_, as indicated by an ion peak [M – H]^−^ at *m*/*z* 375.21784 from its negative HRESIMS. The NMR spectroscopic data of compound **2** showed high similarity with those of compound **1**, except for a hydroxyl group at C-15, which was further identified by the correlations of *δ*_H_ 5.06 (H-17a) and 4.97 (H-17b) with *δ*_C_ 81.1 (C-15) and 41.7 (C-13), and of *δ*_H_ 3.61 (H-15) with *δ*_C_ 34.8 (C-14) in its HMBC spectrum (Figure 3). Other parts of compound **2** were identical to those of **1** by detailed analysis of their MS, NMR, and specific rotation value. Consequently, compound **2** was identified as sigesbeckin B. 

**Figure 4:**
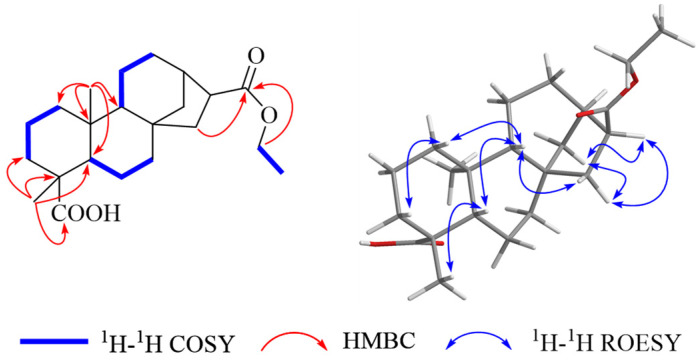
Key HMBC, ^1^H-^1^H COSY, and ^1^H-^1^H ROESY correlations of compound **3**.

The molecular formula of **3** was identified as C_22_H_34_O_4_, with six degrees of unsaturation, by the ion peak [M – H]^−^ at *m*/*z* 361.23832 (calculated for 361.23843) from its HRESIMS spectra. The NMR spectra (Table 1) revealed that compound **3** has the same carbon framework as compound **1** and is similar to 16*α*-H-*ent*-kaurane-17,18-dioic acid, 17-methyl ester (**6**) [31], except for an ethyl ester group replacing the methyl ester at C-17. The difference was further identified by the HMBC correlations of *δ*_H_ 4.11 (H-21) with *δ*_C_ 177.5 (C-17), and the ^1^H-^1^H COSY correlations of *δ*_H_ 4.11 (H-21)/1.26 (H-22). The ROESY spectrum (Figure 4) was utilized to ascertain the relative configuration of compound **3**. The NOE correlations of *δ*_H_ 2.62 (H-16)/1.59 (H-14a)/1.67 (H-15) indicated an *α*-orientation of H-17. The identical absolute configuration of compound **3** as 4*R*, 5*S*, 8*S*, 9*R*, 10*S*, 13*R*, and 16*S* was supported by similar specific rotation values and the same positive signs with compound **6**. Thus, compound **3** was deduced as shown and named sigesbeckin C.

In addition, eight known constituents, 16*α*-hydro-*ent*-kauran-17,19-dioic acid (**4**) [32], 18-hydroxy-kauran-16-*ent*-19-oic acid (**5**) [29], 16*α*-H-*ent*-kaurane-17,18-dioic acid, 17-methyl ester (**6**) [31], *ent*-15*β*-hydroxy-kaur-16-en-19-oic acid (**7**) [33], *ent*-15*β*,17-dihydroxykauran-19-oic acid (**8**) [33], *ent*-16*β*,17-dihydroxykauran-19-oic acid (**9**) [34], *ent*-kuarane-16*α*,17,18-triol (**10**) [35], and 16-*O*-acetyldarutigenol (**11**) [36] were identified by comparing their spectroscopic data with the literature.

The antibacterial bioactivities of compounds **1**–**11** against MRSA and VRE were measured. The results (Table 2) showed that **1** and **5** had certain antibacterial activities on MRSA and VRE with MIC of 64 μg·mL^−1^. In Table 3, data on the synergistic activity of all isolated diterpenes combined with eight antibiotics against MRSA and VRE were provided. The meaning of the fractional inhibitory concentration indices (FICIs) as a synergistic effect (≤0.5) was described previously, of which, compounds **3** and **11** exhibited synergy with doxorubicin hydrochloride (DOX) against MRSA and VRE with FICI values of 0.25 (**3** + DOX, MRSA), 0.5 (**11** + DOX, MRSA), 0.5 (**3** + DOX, VRE), and 0.078 (**11** + DOX, VRE), respectively (Table 3). Both compounds **3** and **5** synergized with vancomycin (VAN) against VRE with an FICI value of 0.3125. Moreover, MRSA was most susceptible to combinations of compound **3** with DOX. Compounds **3**, **5**, and **11** were shown to increase the sensitivity of VAN and DOX in the VRE strain.

The antibacterial properties of compounds **1**–**11** were rigorously evaluated against MRSA and VRE, two prominent multidrug-resistant bacterial strains posing significant challenges to public health. As summarized in Table 2, the assay revealed that compounds **1** and **5** demonstrated certain antibacterial activities against both MRSA and VRE, exhibiting MICs of 64 μg·mL^−1^, suggesting potential utility in the development of new therapeutic strategies to combat these resistant organisms.

The potential enhancement of existing antibiotic therapies was further investigated by examining the synergistic interactions between the isolated diterpenes (compounds **1**–**11**) and eight commonly used antibiotics against MRSA and VRE. The concept of fractional inhibitory concentration indices (FICIs) was utilized to quantitatively evaluate the degree of synergy, with FICIs ≤ 0.5 indicating a synergistic effect, as previously defined [17]. Among the tested combinations, compounds **3** and **11** exhibited notable synergistic activities when combined with doxorubicin hydrochloride (DOX). Specifically, compound **3** demonstrated an FICI of 0.25 against MRSA and 0.5 against VRE when paired with DOX, while compound **11** displayed an FICI of 0.5 against MRSA and an impressive value of only 0.078 against VRE when combined with DOX (Table 3). These findings underscore the potential for these natural products to significantly enhance the efficacy of DOX against drug-resistant bacteria.

Additionally, compounds **3** and **5** demonstrated synergistic effects with VAN, another frontline antibiotic, against VRE, achieving an FICI value of 0.3125. This observation underscores the capacity of these compounds to sensitize VRE to VAN treatment, thereby potentially overcoming resistance mechanisms and restoring the effectiveness of this critical antibiotic. Of particular interest was the heightened susceptibility of MRSA to combinations involving compound 3 and DOX, suggesting a unique interaction that amplifies the antibacterial action against this difficult-to-treat pathogen. Moreover, compounds 3, 5, and 11 collectively enhanced the sensitivity of both MRSA and VRE strains to VAN and DOX, indicating their broad potential as adjuvants in combination therapy regimens aimed at mitigating antibiotic resistance. These results pave the way for further investigation into the molecular basis of these interactions and the possible development of novel therapeutic strategies incorporating natural products to combat antibiotic-resistant infections.

## 3. Materials and Methods

### 3.1. General Experimental Procedures

Optical rotation measurements were conducted using an Autopol IV automatic polarimeter (Rudolph Research Analytical, Hackettstown, NJ, USA). The instrument was calibrated with standard solutions prior to each measurement session. Samples were dissolved in appropriate solvents (e.g., methanol or chloroform) to achieve concentrations suitable for optical activity determination, typically ranging from 0.1 to 3.0 mg/mL. Measurements were performed at a wavelength of 589 nm at room temperature (25 °C), and the specific rotation values are reported as degrees per centimeter per gram. 

Infrared spectra were recorded on a NICOLET iS10 Fourier transform infrared spectrophotometer (Thermo Fisher Scientific, Waltham, MA, USA). KBr pellets were prepared by grinding approximately 1–2 mg of the sample with anhydrous potassium bromide (~100 mg) and pressing the mixture into a transparent pellet under vacuum. Spectra were acquired over a wavenumber range of 4000 to 400 cm^−1^ at a resolution of 4 cm^−1^, averaging multiple scans (typically 64) to enhance the signal-to-noise ratio. 

NMR spectra were obtained using a Bruker AVANCE NEO 400 MHz spectrometer equipped with a 5 mm broadband observe (BBO) probe (Bruker, Billerica, MA, USA). Samples were dissolved in deuterated solvents, such as CDCl_3_ or DMSO-*d*_6_, and chemical shifts (*δ*) are reported in parts per million (ppm) relative to tetramethylsilane (TMS) as an internal reference standard. Standard pulse sequences were utilized for ^1^H and ^13^C NMR experiments, including DEPT, COSY, NOESY, HSQC, and HMBC for structure elucidation. TopSpin 4.0.9 (Bruker) was employed to process free induction decays (FIDs) and to perform advanced data manipulations, such as baseline correction, peak picking, and integration. 

HRESIMS analyses were carried out on an Agilent 1290 UPLC coupled to a 6545 Q-TOF mass spectrometer. The system was operated in both positive and negative ionization modes, with optimized conditions for each compound class. Data were acquired over a mass range of *m*/*z* 100–1700, and accurate mass measurements were achieved using an external calibration mixture provided by Agilent. MassHunter Qualitative Navigator B.08.00 (Agilent Technologies, Santa Clara, CA, USA) was employed for high-throughput data processing, enabling efficient data extraction, qualitative analysis, and quantitative analysis.

Semi-preparative HPLC was conducted on an Agilent 1260 liquid chromatograph, fitted with a X-Bridge C18 column (250 mm × 9.4 mm I.D., particle size 5 µm). Gradient elution programs were designed based on the chemical properties of the compounds, utilizing solvent systems, like water–acetonitrile or water–methanol. Flow rates were typically set between 2.5 and 3.0 mL/min, and UV detection was employed at multiple wavelengths (195, 210 and 254 nm) to monitor eluent absorbance.

Chromatographic purification involved the use of silica gel (Qingdao Marine Chemical Inc., Qingdao, China; particle size 200–300 mesh), C-18 reversed-phase silica gel (Daiso Co., Tokyo, Japan; particle size 40–60 µm), and Sephadex LH-20 (Amersham Pharmacia Biotech, Stockholm, Sweden). Flash column chromatography was performed under gradient elution conditions, with mobile phases tailored to the polarity of the compounds being separated.

TLC was performed on pre-coated silica gel plates (GF_254_, Qingdao Haiyang Chemical Co., Ltd., Qingdao, China), with spots visualized under UV light (254 and 365 nm) and/or developed using appropriate spray reagents, such as vanillin–sulfuric acid for phenolics. Rf values were calculated and compared against standards for initial assessment of compound purity. Additionally, a sulfuric acid–ethanol solution (10% H_2_SO_4_ in EtOH) was used as a universal spray reagent to visualize a wide range of organic compounds. After application, plates were heated at 110 °C until colored spots appeared. This method is particularly useful for detecting compounds that do not fluoresce under UV light or fail to react with other common spray reagents. 

The OD_600nm_ values were measured using an enzyme label instrument (SPECTRA MAX 190, MDC, Frederick, MD, USA). All antibiotics utilized in the study were sourced from Macklin Reagent, located in Shanghai, China. Bacterial cultures were cultivated in tryptic soy broth (TSB) medium to ensure optimal growth conditions. Throughout the experiment, all solvents employed were of analytical grade, guaranteeing the highest level of purity and consistency in the results obtained.

### 3.2. Plant Materials

The stems and leaves of *S. orientalis* were collected in May 2021 from Lijiang City (Yunnan Province, China) and taxonomically identified by Dr. Jun Zhang at Kunming Plant Taxonomy Biotechnology Co., Ltd., Kunming, China. A voucher specimen (Luo202210520) has been deposited at Yunnan University.

### 3.3. Extraction and Isolation

The pulverized aerial parts of *S. orientalis* (20 kg) were subjected to reflux extraction with 90% ethanol (80 L × 3, 5 h each). The extract was evaporated under reduced pressure at 55.0 °C, yielding 1.35 kg of crude extract. The residue (1.35 kg) was extracted with ethyl acetate (4 × 10 L), yielding an ethyl acetate fraction (603 g). This fraction was later separated into four distinct fractions (Fr. A–Fr. D) through the utilization of silica gel column chromatography, employing a gradient system consisting of petroleum ether and ethyl acetate in varying proportions (ranging from 10:0 to 0:1, *v*/*v*). 

Fr. B (41.0 g) was subjected to silica gel column chromatography using a chloroform/ethyl acetate gradient (20:1 to 0:1, *v*/*v*), yielding four subfractions, namely Fr. B1–Fr. B4. Fr. B1 (13.3 g) was further purified using MPLC (RP-C18, methanol/water, 25%→100%, *v*/*v*, 25 mL/min), followed by Sephadex LH-20 gel chromatography (methanol), yielding compounds **1** (101.0 mg), **4** (107.0 mg), **7** (340.0 mg), and **8** (110.0 mg). Fr. B2 (9.2 g) was further purified using silica gel column chromatography (chloroform/ethyl acetate, 9:1, *v*/*v*), yielding compounds **5** (1.3 g) and **9** (339.0 mg). Fr. B3 (12.0 g) was purified using Sephadex LH-20 gel chromatography with methanol (Sigma-Aldrich, St. Louis, MO, USA), followed by silica gel column chromatography (petroleum ether/acetone, 5:1, *v*/*v*), yielding compounds **6** (78.0 mg) and **11** (445.0 mg).

Fr. D (45.0 g) was fractionated using MPLC with MCI as the stationary phase and a methanol/water gradient (3:10 to 1:0, *v*/*v*), resulting in three subfractions, namely Fr. D1–Fr. D3 and compound **10** (128.0 mg). Compound **2** (228.0 mg) was obtained by purifying Fr. D1 (11.5 g) using silica gel column chromatography with a petroleum ether/ethyl acetate gradient (10:1 to 6:1, *v*/*v*). Fr. D2 (22.5 g) was isolated using MPLC (RP-C18, methanol/water, 55% to 90%, *v*/*v*, 25 mL/min) and subsequently purified via Sephadex LH-20 gel chromatography to yield Fr. D2-1 (3.3 g). Compound **3** (110.0 mg) was obtained from Fr. D2-1 by using HPLC (acetonitrile/water, 78%→100%, *v*/*v*, 2.5 mL/min, TR = 10.5 min).

Sigesbeckin A (**1**): white powder; HRMS (ESI) *m*/*z*: 383.21942, (calcd. for C_22_H_32_O_4_, [M + Na]^+^, 383.21928) ${\left[\alpha \right]}_{D}^{25.0}$ −191.2 (*c* 0.45, CH_3_OH); IR (KBr) *V*_max_ 2971, 2963, 2927, 1746, 1442, 1381, 1230, 1176, 1069, and 1053 cm^−1^; 1D NMR spectral data are provided in Table 1.

Sigesbeckin B (**2**): white powder; HRMS (ESI) *m*/*z* 375.21784 (calcd. for C_22_H_32_O_5_ [M–H]^−^, 375.21770); ${\left[\alpha \right]}_{D}^{25.0}$ −74.2 (*c* 2.03, CH_3_OH); IR (KBr) *V*_max_ 3672, 2985, 2972, 2902, 1743, 1450, 1393, 1381, 1230, 1067, 1053, and 896 cm^−1^; 1D NMR spectral data are provided in Table 1.

Sigesbeckin C (**3**): white powder; HRMS (ESI) *m*/*z* 361.23832 (calcd. for C_22_H_34_O_5_ [M − H]^−^, 361.23843); ${\left[\alpha \right]}_{D}^{25.0}$ −85.7 (*c* 1.36, CH_3_OH); IR (KBr) *V*_max_ 3726, 2973, 2955, 2912, 1731, 1669, 1407, 1392, 1374, 1259, 1184, 1164, and 1052 cm^−1^; 1D NMR spectral data are provided in Table 1.

### 3.4. Determination of Antimicrobial Activities

The MICs of compounds (**1**–**11**) and antibiotics were determined using the broth microdilution method in accordance with the 2020 guidelines established by the Clinical and Laboratory Standards Institute (CLSI), as described in the existing scientific literature [5,15] (refer to the Supplementary Materials for further information).

### 3.5. Assessment of Synergistic Effects

The synergistic effects of compounds (**1**–**11**) combined with eight antibiotics were detected by the checkerboard method [5] (refer to the Supplementary Materials for further information).

## 4. Conclusions

In our investigation of *S. orientalis*, we successfully isolated three novel *ent*-kaurane diterpenes in addition to identifying eight previously known analogs. Notably, compounds **1** and **5** displayed moderate antibacterial capabilities against MRSA and VRE, indicating their potential as antimicrobial agents. Further exploration into synergistic interactions revealed that compounds **3**, **5**, and **11** significantly enhanced the efficacy of DOX and VAN, demonstrating potent synergistic activities with these antibiotics against both resistant strains. This discovery underscores the promising role of these diterpenes in augmenting current antibiotic therapies, thereby positioning them as valuable candidates for future development into innovative therapeutic strategies to tackle drug-resistant infections.

## Figures and Tables

**Table 1:** ^13^C (100 MHz) and ^1^H (400 MHz) NMR Spectral Data for **1**–**3**.

	1 ^a^		2 ^b^		3 ^b^	
No.	*δ*_C_, Type	*δ*_H_ (Mult., *J* in Hz)	*δ*_C_, Type	*δ*_H_ (Mult., *J* in Hz)	*δ*_C_, Type	*δ*_H_ (Mult., *J* in Hz)
1	40.7, t	Ha: 1.51, m	39.7, t	Ha: 1.82, m	40.7, t	Ha: 1.88, m
		Hb: 1.43, m		Hb: 0.76, m		Hb: 0.83, m
2	18.4, t	1.56, m	17.9, t	Ha: 1.51, overlap	18.7, t	Ha: 1.67, m
				Hb: 1.33, m		Hb: 1.54, m
3	32.3, t	Ha: 2.31, m	32.3, t	Ha: 1.51, overlap	37.8, t	Ha: 2.17, m
		Hb: 1.06, m		Hb: 1.36, m		Hb: 1.03, m
4	47.5, s		46.7, s		43.8, s	
5	52.3, d	1.28, m	51.0, d	1.21, m	56.9, d	1.07, m
6	21.8, t	1.74, m	20.8, t	Ha: 1.67, m	22.4, t	1.86, m
				Hb: 1.59, overlap		
7	40.1, t	Ha: 1.91, m	35.6, t	Ha: 1.59, overlap	38.0, t	Ha: 1.87, m
		Hb: 0.82, m		Hb: 1.27, overlap		Hb: 1.25, m
8	43.9, s		47.0, s		45.1, s	
9	55.1, d	1.10, m	53.0, d	0.97, m	55.1, d	1.02, m
10	39.4, s		39.1, s		39.6, s	
11	18.3, t	1.82, m	18.1, t	Ha: 1.84, overlap	19.1, t	Ha: 1.89, m
				Hb: 1.40, m		Hb: 1.45, m
12	33.0, t	Ha: 1.60, m	31.9, t	Ha: 2.12, m	31.1, t	1.53, m
		Hb: 1.48, m		Hb: 1.02, m		
13	43.8, d	2.64, m	41.7, d	2.63, m	41.1, d	2.47, m
14	39.6, t	Ha: 1.95, m	34.8, t	Ha: 1.59, overlap	41.0, t	Ha: 1.59, m
		Hb: 1.14, m		Hb: 1.27, overlap		Hb: 1.47, m
15	48.8, t	2.05, overlap	81.1, d	3,61, s	44.6, d	1.67, m
16	155.5, s		159.6, s		45.5, d	2.62, m
17	103.2, d	4.79, d, *J* = 24.0	107.6, d	Ha: 5.06, s	177.5, s	
				Hb: 4.97, s		
18	72.1, t	Ha: 4.01, d, *J* = 10.5	71.3, t	Ha: 4.36, d, *J* = 10.3	28.9, q	1.25, s
		Hb: 3.49, d, *J* = 10.5		Hb: 3.93, d, *J* = 10.3		
19	181.8, s		175.9, s		184.0, s	
20	15.5, q	0.99, s	15.5, q	0.92, s	15.5, q	0.93, s
21	171.0 s		170.1 s		60.3, t	4.11, q
22	20.8, q	2.06, s	20.6, q	1.99, s	14.3, q	1.26, s

^a^ Measured in CDCl_3_; ^b^ measured in DMSO-*d_6_*.

**Table 2:** The MIC values of compounds **1**–**11** against MRSA and VRE.

Compounds	MIC (μg·mL^−1^)	
	MRSA	VRE
**1**	64	64
**2**	256	256
**3**	256	256
**4**	256	256
**5**	64	256
**6**	256	256
**7**	256	256
**8**	256	256
**9**	256	256
**10**	256	256
**11**	256	256
Ampicillin (AMP)	64	0.5
Vancomycin (VAN)	1	512
Doxorubicin hydrochloride (DOX)	16	128

**Table 3:** FICIs were determined using a checkerboard assay to assess the interaction between diterpenes and antibiotics against MRSA and VRE.

Strains	Agents	MIC in Combination (μg·mL^−1^)		FICI
MRSA	**3** + DOX	64	2	0.375
	**3** + DOX	32	4	0.375
	**3** + DOX	32	2	0.25
	**11** + DOX	64	4	0.5
VRE	**3** + DOX	64	32	0.5
	**3** + VAN	16	256	0.3125
	**5** + VAN	16	64	0.3125
	**11** + DOX	64	8	0.3125

## Data Availability

Data are contained within the article.

## Supplementary Materials

The following supporting information can be downloaded at: https://www.mdpi.com/article/10.3390/molecules29194631/s1. The general experimental procedures, biological assay, and original MS, IR, ORD, and NMR spectra of **1**–**3** (PDF).
