# Supplementary material for: Three New Ent-Kaurane Diterpenes with Antibacterial Activity from Sigesbeckia orientalis
Source: Molecules. 2024 Sep 29;29(19):4631. doi: 10.3390/molecules29194631 (PMC11477649; doi:10.3390/molecules29194631)
Supplement: Supplementary file 1 [file molecules-29-04631-s001.zip › molecules-3217408-supplementary.pdf]

Supplementary Materials

Three New *Ent*-Kaurane Diterpenes with Antibacterial Activity from *Sigesbeckia orientalis*

Zhong-Shun Zhou [1], Zhao-Jie Wang [1], Bei Tian [1], Yan-Yan Zhu [1], Mei-Zhen Wei [1], Yun-Li Zhao [1] and Xiao-Dong Luo [1,2,*]

[1] Yunnan Characteristic Plant Extraction Laboratory, Key Laboratory of Medicinal Chemistry for Natural Resource, Ministry of Education and Yunnan Province, School of Chemical Science and Technology, Yunnan University, Kunming 650091, China; zhouzhongshun195@outlook.com (Z.-S.Z.); wangzhaojie10111@163.com (Z.-J.W.); 15050597099@163.com (B.T.); 22021016018@mail.ynu.edu (Y.-Y.Z.); 22021016017@mail.ynu.edu.cn (M.-Z.W.); zhaoyunli@mail.kib.ac.cn (Y.-L.Z.)

[2] State Key Laboratory of Phytochemistry and Plant Resources in West China, Kunming Institute of Botany, Chinese Academy of Sciences, Kunming 650201, China

* Correspondence: xdluo@ynu.edu.cn; Tel.: +86-0871-65032908

Table of Contents

1. Determination of antimicrobial activities........ - 1 -

2. Assessment of Synergistic Effects .... - 1 -

3. NMR, HRESIMS, IR and ORD of sigesbeckin A (**1**) .... - 3 -

4. NMR, HRESIMS, IR and ORD of sigesbeckin B (**2**) .... - 8 -

5. NMR, HRESIMS, IR and ORD of sigesbeckin C (**3**)........ - 13 -

1. Determination of antimicrobial activities

The MICs of compounds and antibiotics were determined using the broth microdilution method according to the Clinical and Laboratory Standards Institute (CLSI) 2020 guidelines. According to the reported literature method[1]. The bacterial solution in the logarithmic growth phase was standardized to 1 × 10^5 CFU/mL, and a stock solution of the compound and antibiotic was prepared in dimethyl sulfoxide (DMSO), using the adjusted bacterial suspension to perform continuous double dilutions of the test drug in a 96-well plate, ranging from 512 μg/mL to 1 μg/mL. Vancomycin (VAN) and ampicillin (AMP) were used as controls. The MIC value was defined as the lowest concentration at which no visible live bacteria were observed at 37°C for 18-24 h. All tests were performed three times.

2. Assessment of Synergistic Effects

Firstly, detect the MIC value of each antibiotic. The checkerboard method is to double dilute the antibiotics in the TSB medium so that the concentration of antibiotic is distributed between 1/16 × MIC and 1/2 × MIC, that is, antibiotic solution (10 μL) was added to each row of the 96-well plate according to the decreasing order of concentration, and each concentration was repeated for 6 wells. In the same way, the synergistic compound solution was added into each column of the 96-well plate successively from high to low concentration, and 6 wells were repeated in each group. Then 80 μL bacterial solution (105-106 CFU/mL) was added to each well. After the whole synergetic solution was

prepared, the 96-well plates were cultured in an incubator at 37℃ for 18 h. Finally, the absorbance of each well and the MIC value of each drug combined were determined (OD at 600 nm), fractional inhibitory concentration index (FICI) = MICA in combination/MICA alone + MICB in combination/MICB alone, FICI ≤ 0.5 synergistic, 0.5 < FICI ≤ 1 additive.

Table S1 The MIC values of 8 antibiotics against MRSA and VRE

Compounds	MIC（$\mu g \cdot mL^{-1}$）	
	MRSA	VRE
**Ampicillin**	64	0.5
**Doxorubicin HCL**	16	128
**Lincomycin**	1	1024
**Vancomycin**	1	512
**Streptomycin**	4	2048
**Tetracyclin HCl**	0.125	0.25
**Chloramphenicol**	4	128
**Norfloxacin**	1	1
**Azithromycin**	2	1024

3. NMR, HRESIMS, IR and ORD of sigesbeckin A (1)

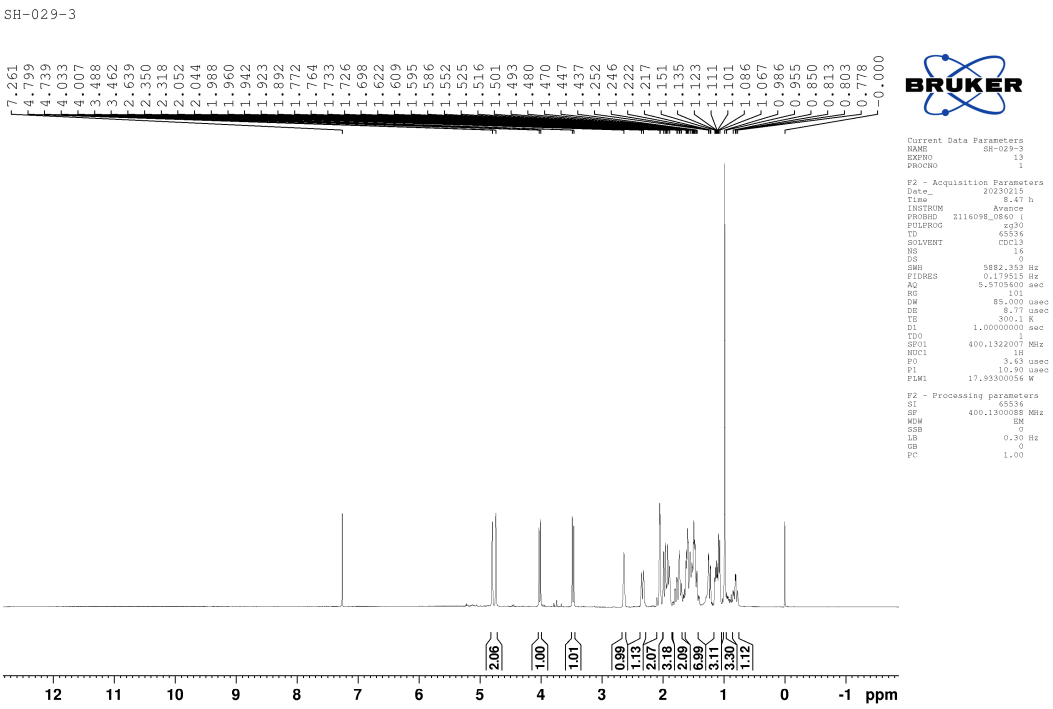

Figure S1. ^{1}H NMR (400 MHz) spectrum of sigesbeckin (**1**) in $CDCl_3$.

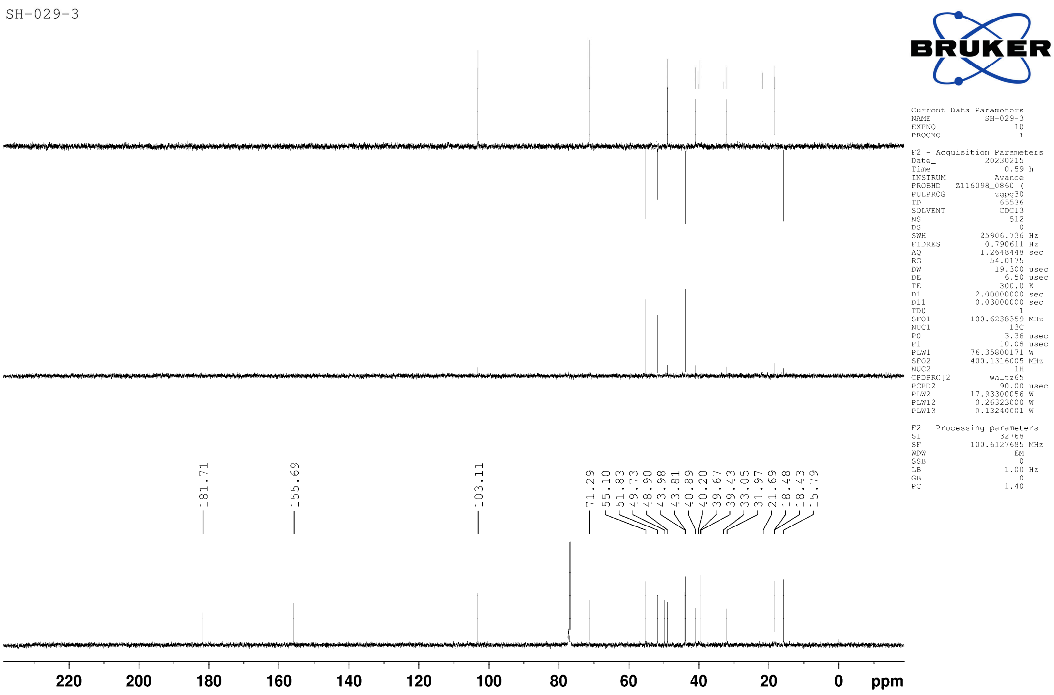

Figure S2. ^{13}C NMR (100 MHz) and DEPT spectra of sigesbeckin A (**1**) in $CDCl_3$.

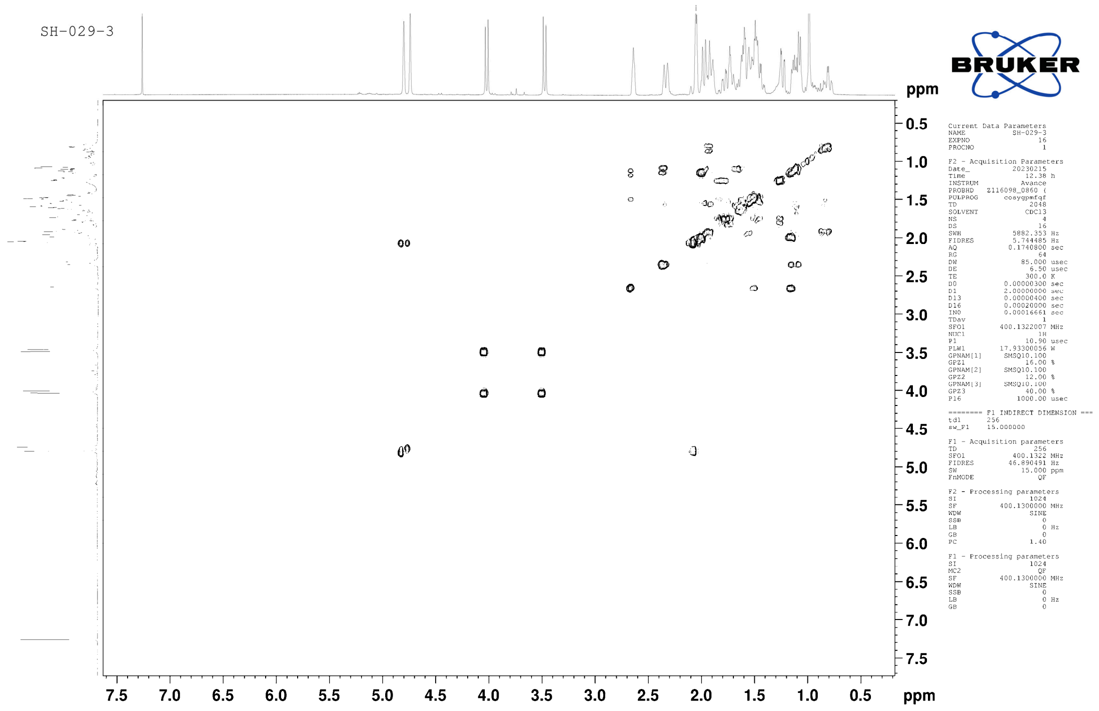

Figure S3. ^1H-^1H COSY spectrum of sigesbeckin A (**1**) in $CDCl_3$.

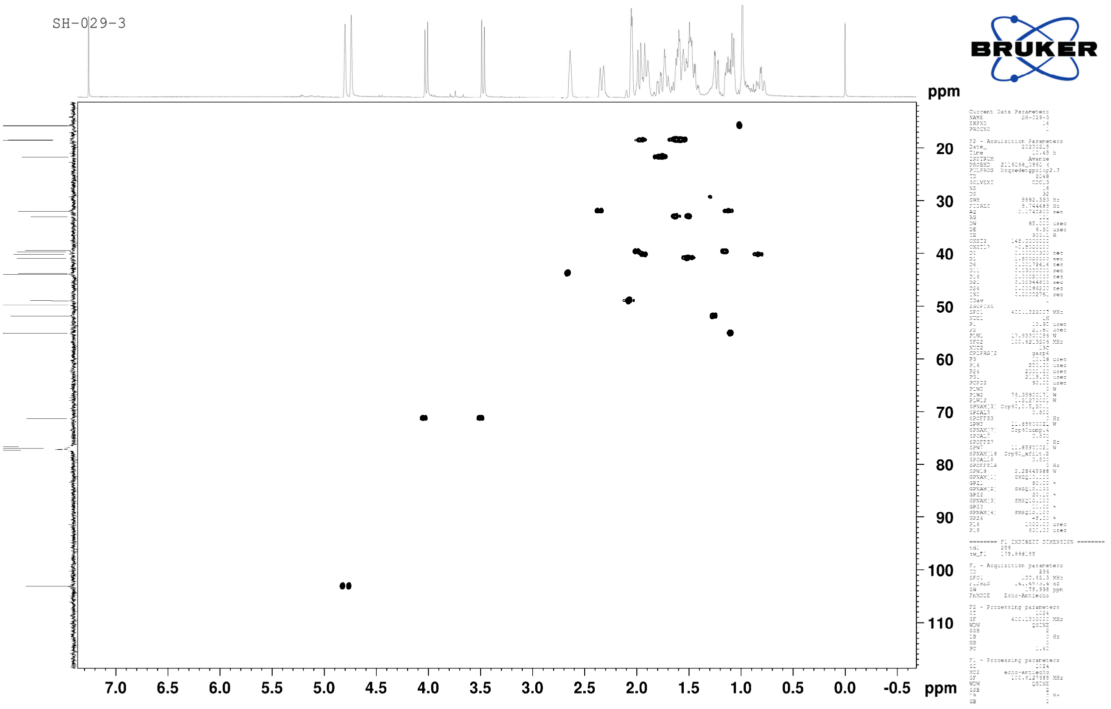

Figure S4 HSQC spectrum of sigesbeckin A (**1**) in $CDCl_3$.

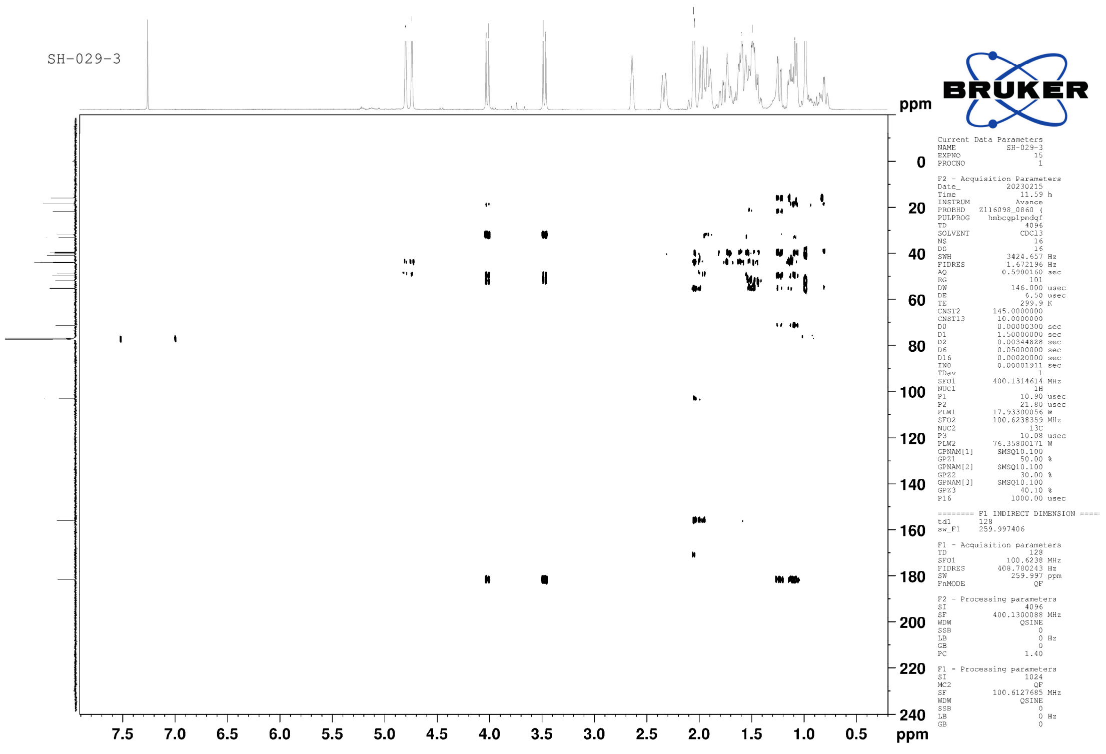

Figure S5. HMBC spectrum of sigesbeckin A (**1**) in $CDCl_3$.

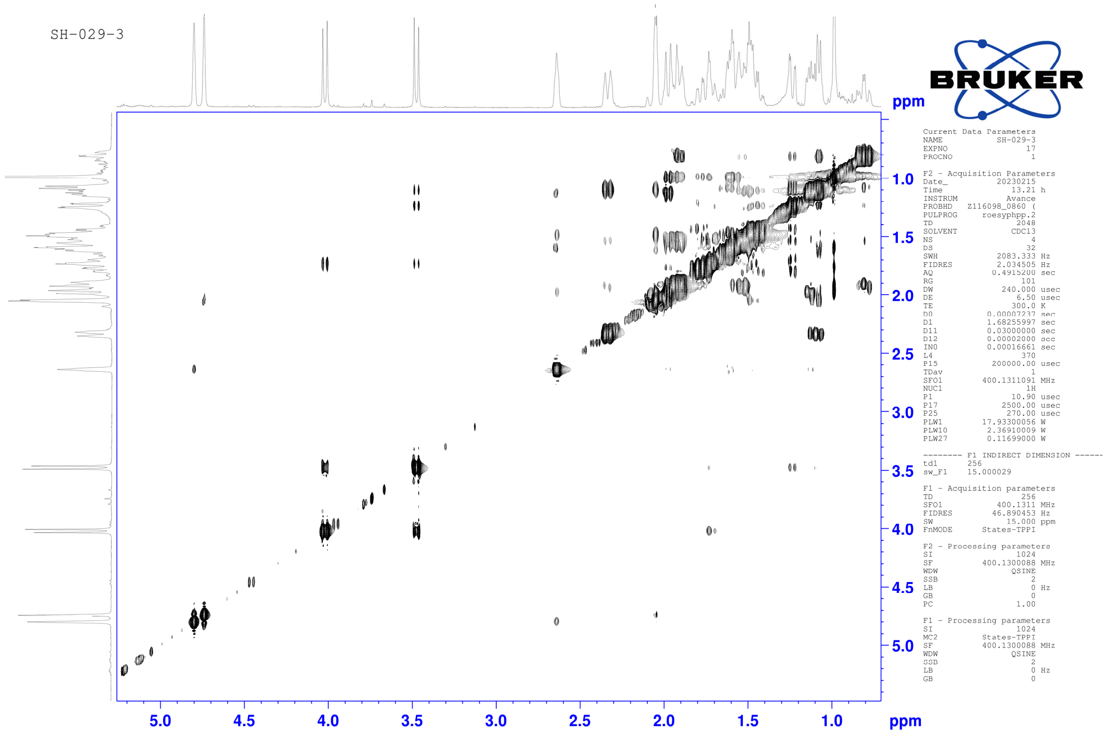

Figure S6. ^1H-^1H ROESY spectrum of sigesbeckin A (**1**) in $CDCl_3$.

Qualitative Analysis Report

**Data File**	SH-029-2.d	**Sample Name**	SH-029-P2-2
**Sample Type**	Sample	**Position**	P1-A3
**Instrument Name**	Instrument 1	**User Name**	
**Acq Method**	20220426-liangtong-P.m	**Acquired Time**	2024/8/8 20:24:53 (UTC+08:00)
**IRM Calibration Status**	Success	**DA Method**	Default.m
**Comment**			
**Sample Group**		**Info.**	
**Stream Name**	LC 1	**Acquisition Time (Local)**	2024/8/8 20:24:53 (UTC+08:00)
**Acquisition SW Version**	6200 series TOF/6500 series Q-TOF B.09.00 (B9044.0)	**QTOF Driver Version**	8.00.00
**QTOF Firmware Version**	25.723	**Tune Mass Range Max.**	3200

Spectra

**Fragmentor Voltage**	**Collision Energy**	**Ionization Mode**
120	0	ESI

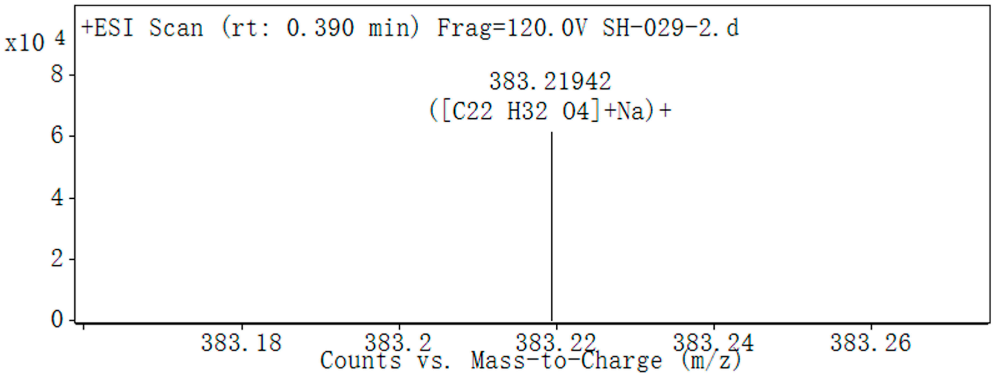

Spectrum Identification Results: + Scan (rt: 0.390 min) (SH-029-2.d)

Best	ID Sourc	Nam	Formul	Specie	m/z	Scor	Diff (ppm	Score (MFG
☐	**MFG**		**C22 H32 O4**	**(M+Na)+**	**383.2194**	**49.02**	**-0.14**	**98.03**

Species	Lib/D	m/z	Score (iso. abund	RT	Score (mass	RT Dif	Score (MFG, MS/M	Score (MS	Score (MFG	Score (iso. spacing	Heigh	Ion Formul
(M+Na)+		383.2194	94.18		99.98			98.03	98.03	98.75	61730.5	C22 H32 Na O

Height (Calc)	Height Sum% (Cal	Height % (Calc	m/z (Calc	Diff (mDa	Heigh	Height	Height Sum	m/z	Diff (ppm
60194.3	78.1	100	383.21928	-0.1	61730.5	100	80.1	383.2194	-0.36
14636.2	19	24.3	384.22269	0	13199.7	21.4	17.1	384.2226	0.05
2196.7	2.9	3.6	385.22552	2	2097	3.4	2.7	385.2234	5.27

--- End Of Report ---

Figure S7. HRESIMS of sigesbeckin A (**1**).

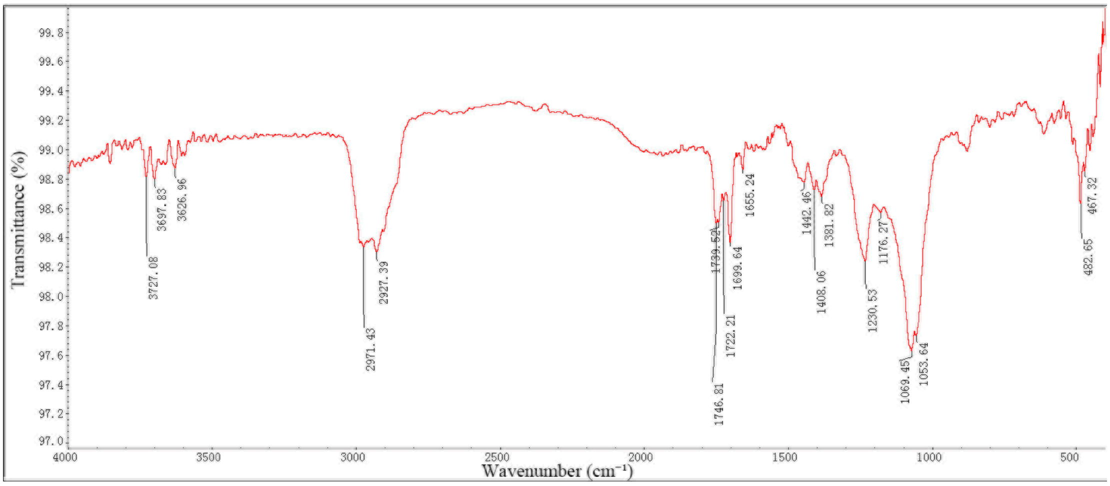

Figure S8. IR spectrum of sigesbeckin A (**1**).

Rudolph Research Analytical

This sample was measured by Autopol IV, Serial Number: 83650

Manufactured by Rudolph Research Analytical, Hackettstown, NJ, USA.

Measurement Date : 2024/8/7

Method Name : Specific Rotation @25C

Set Temperature : 25.0°C

Time Delay : 10

Delay between measurement : 1 Sec

N	Avg.	Std.Dev.	%RSD	Min	Max
5	-191.200	0.795	-0.41	-191.556	-189.778

S.No	Sample ID	Time	Result	Scale	OR °Arc	WLG.nm	Lg.mm	Conc.g/100mL	Temp
1	SH-029	17:56:24	-189.778	SR	-0.0854	589	100	0.045	25.0°C
2	SH-029	17:56:31	-191.556	SR	-0.0862	589	100	0.045	25.0°C
3	SH-029	17:56:38	-191.556	SR	-0.0862	589	100	0.045	25.0°C
4	SH-029	17:56:45	-191.556	SR	-0.0862	589	100	0.045	25.0°C
5	SH-029	17:56:52	-191.556	SR	-0.0862	589	100	0.045	25.0°C

Page 1 of 1

Figure S9. Experimental ORD sigesbeckin A (**1**).

4. NMR, HRESIMS, IR and ORD of sigesbeckin B (2)

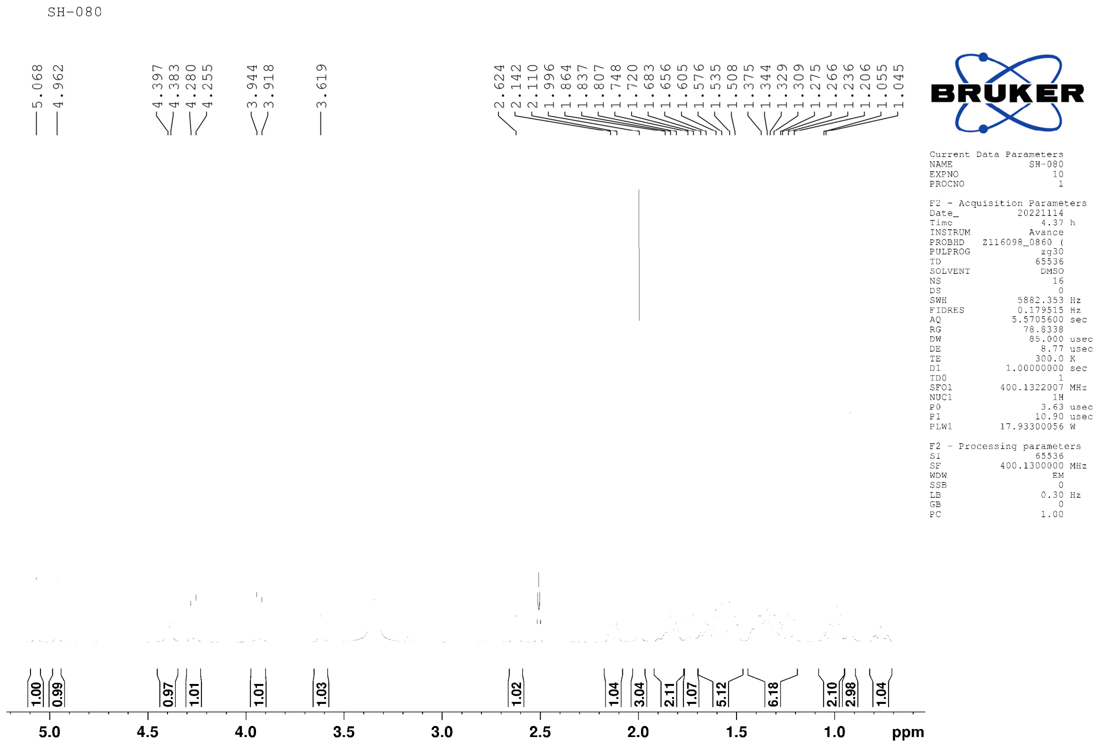

Figure S10. ^{1}H NMR (400 MHz) spectrum of sigesbeckin B (**2**) in DMSO-d_6.

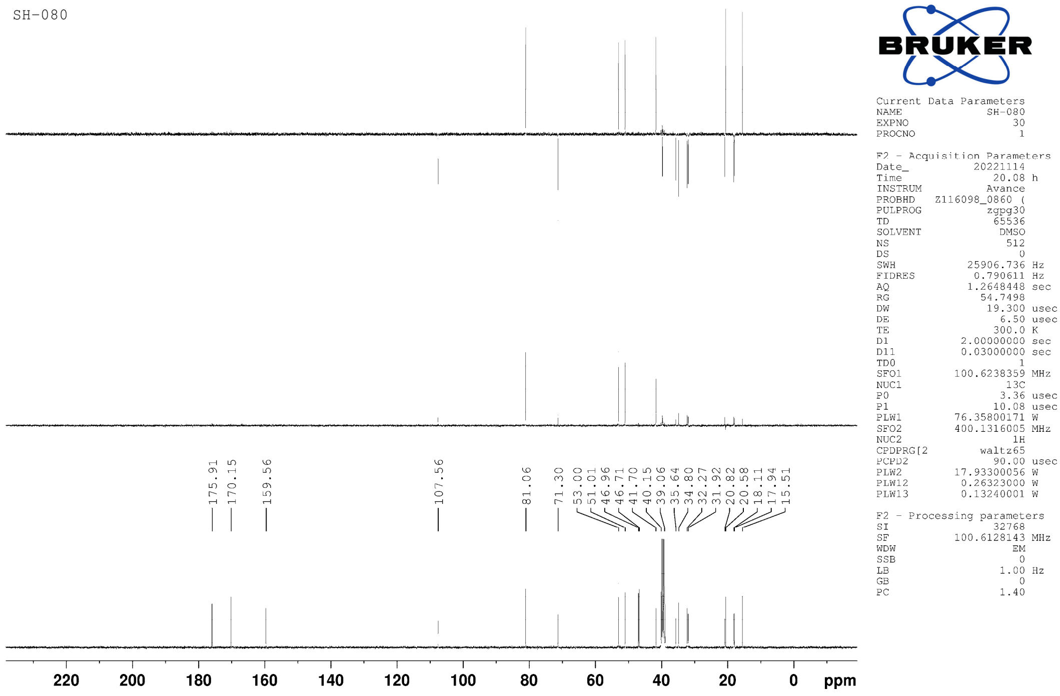

Figure S11. ^{13}C NMR (100 MHz) and DEPT spectra of sigesbeckin B (**2**) in DMSO-d_6.

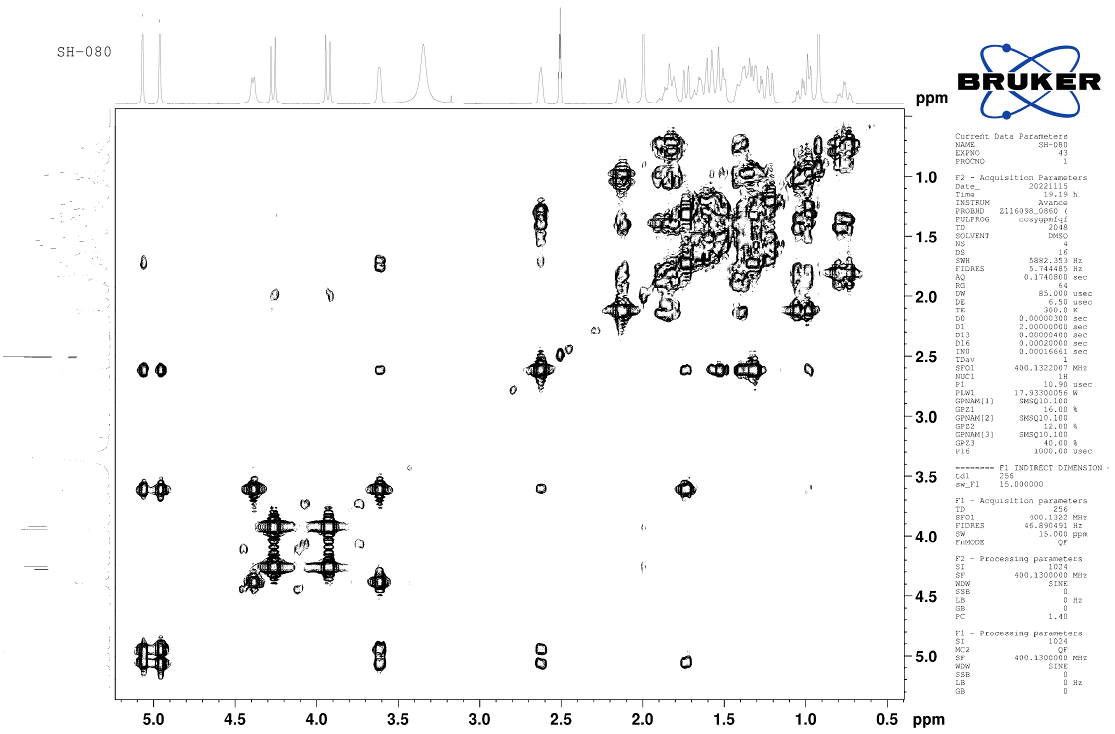

Figure S12. ^{1}H-^{1}H COSY spectrum of sigesbeckin B (**2**) in DMSO-*d*$_6$.

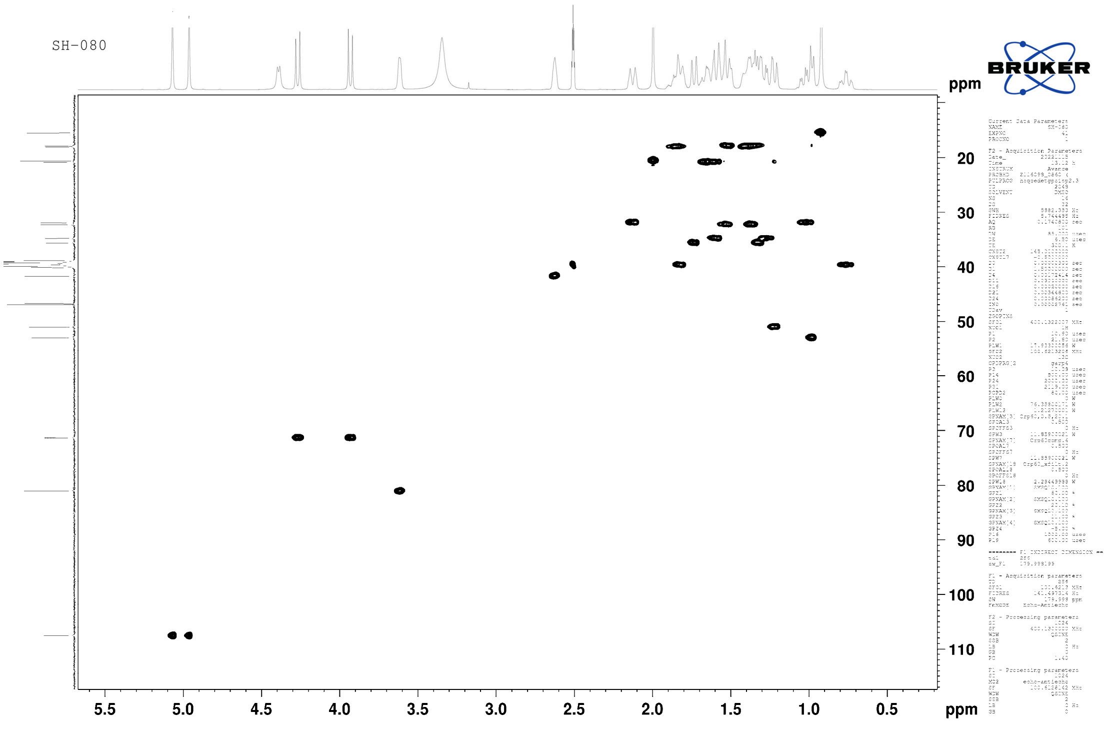

Figure S13 HSQC spectrum of sigesbeckin B (**2**) in DMSO-*d*$_6$.

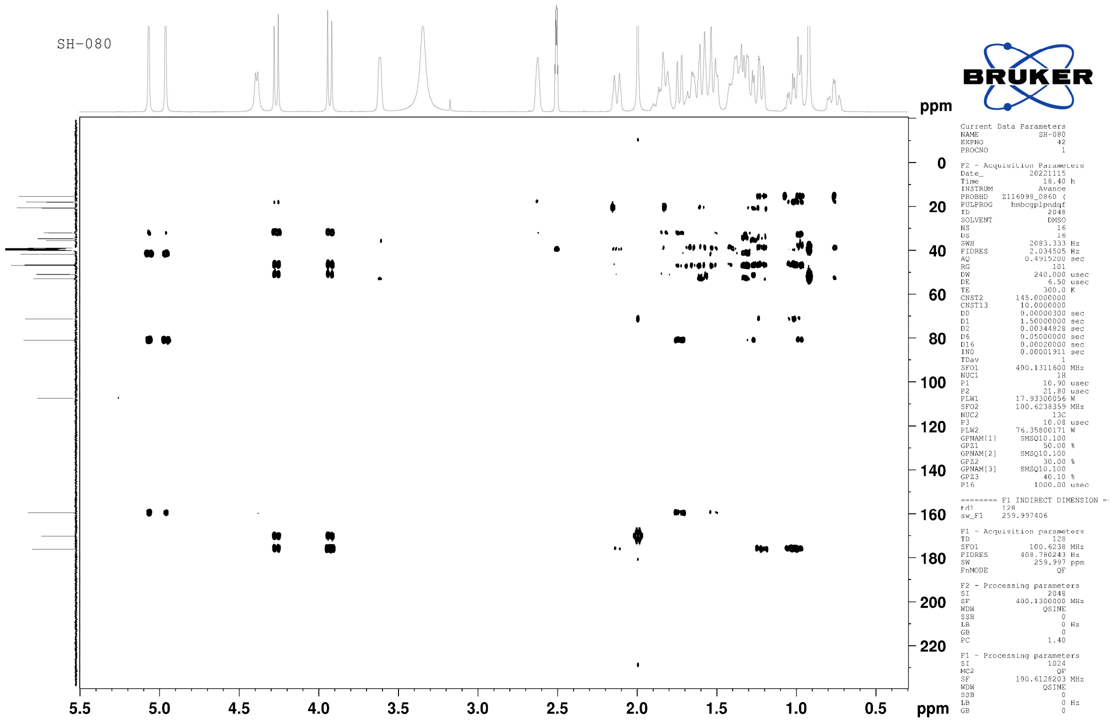

Figure S14. HMBC spectrum of sigesbeckin B (**2**) in DMSO-*d*₆.

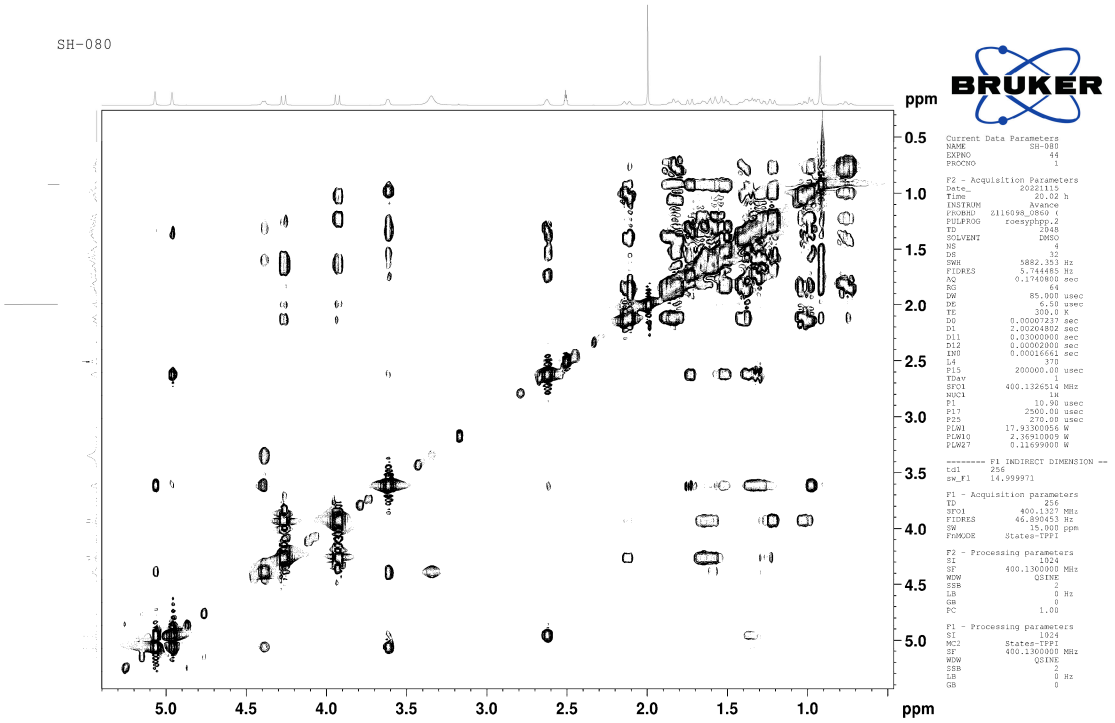

Figure S15. ^{1}H-^{1}H ROESY spectrum of sigesbeckin B (**2**) in DMSO-*d*$_6$.

Qualitative Analysis Report

**Data File**	SH-80-N.d	**Sample Name**	SH-80-N
**Sample Type**	Sample	**Position**	P1-A1
**Instrument Name**	Instrument 1	**User Name**	
**Acq Method**	20220426-liangtong-N.m	**Acquired Time**	2022/11/14 11:38:48 (UTC+08:00)
**IRM Calibration Status**	Success	**DA Method**	Default.m
**Comment**			
**Sample Group**		**Info.**	
**Stream Name**	LC 1	**Acquisition Time (Local)**	2022/11/14 11:38:48 (UTC+08:00)
**Acquisition SW Version**	6200 series TOF/6500 series Q-TOF B.09.00 (B9044.0)	**QTOF Driver Version**	8.00.00
**QTOF Firmware Version**	25.723	**Tune Mass Range Max.**	3200

Spectra

Fragmentor Voltage	Collision Energy	Ionization Mode
120	0	ESI

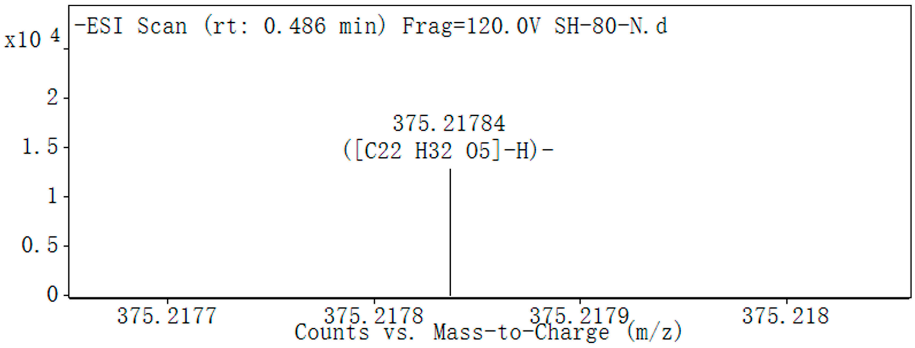

Spectrum Identification Results: - Scan (rt: 0.486 min) (SH-80-N.d)

Best	ID Sourc	Nam	Formul	Specie	m/z	Scor	Diff (ppm	Score (MFG
☐	**MFG**		**C22 H32 O5**	**(M-H)-**	**375.2178**	**49.51**	**-0.73**	**99.01**

Species	Lib/D	m/z	Score (iso. abund	RT	Score (mass	RT Dif	Score (MFG, MS/M	Score (MS	Score (MFG	Score (iso. spacing	Heigh	Ion Formul
(M-H)-		375.2178	98.88		99.55			99.01	99.01	98.09	12883.8	C22 H31 O5

Height (Calc)	Height Sum% (Cal	Height % (Calc	m/z (Calc	Diff (mDa	Heigh	Height	Height Sum	m/z	Diff (ppm
12883.8	78	100	375.2177	-0.1	12764.2	100	77.3	375.2178	-0.37
3136.1	19	24.3	376.2211	-0.6	3141.1	24.6	19	376.2216	-1.49
497.5	3	3.9	377.22383	-1.6	612	4.8	3.7	377.2254	-4.25

--- End Of Report ---

Figure S16. HRESIMS of sigesbeckin B (**2**).

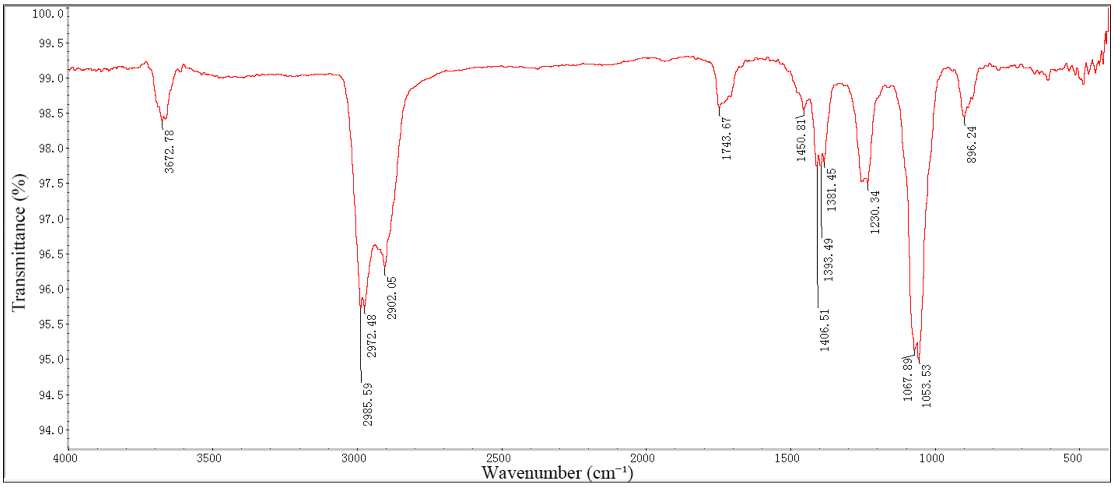

Figure S17. IR spectrum of sigesbeckin B (**2**).

Rudolph Research Analytical

This sample was measured by Autopol IV, Serial Number: 83650

Manufactured by Rudolph Research Analytical, Hackettstown, NJ, USA.

Measurement Date : 2024/8/7

Method Name : Specific Rotation @25C

Set Temperature : 25.0°C

Time Delay : 10

Delay between measurement : 1 Sec

N	Avg.	Std.Dev.	%RSD	Min	Max
5	-74.187	0.000	0.00	-74.187	-74.187

S.No	Sample ID	Time	Result	Scale	OR °Arc	WLG.nm	Lg.mm	Conc.g/100mL	Temp
1	SH-080	18:18:51	-74.187	SR	-0.1506	589	100	0.203	25.0°C
2	SH-080	18:18:58	-74.187	SR	-0.1506	589	100	0.203	25.0°C
3	SH-080	18:19:05	-74.187	SR	-0.1506	589	100	0.203	25.0°C
4	SH-080	18:19:12	-74.187	SR	-0.1506	589	100	0.203	25.0°C
5	SH-080	18:19:19	-74.187	SR	-0.1506	589	100	0.203	25.0°C

Page 1 of 1

Figure S18. Experimental ORD sigesbeckin B (**2**).

5. NMR, HRESIMS, IR and ORD of sigesbeckin C (3)

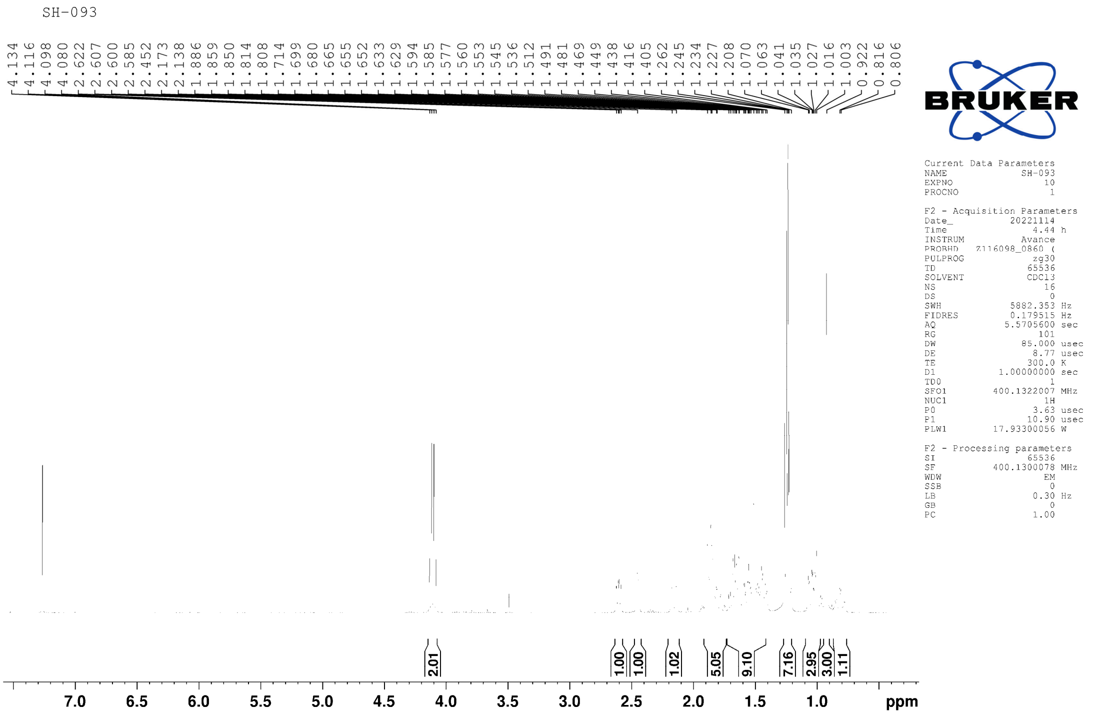

Figure S19. ^1H NMR (400 MHz) spectrum of sigesbeckin C (**3**) in $CDCl_3$.

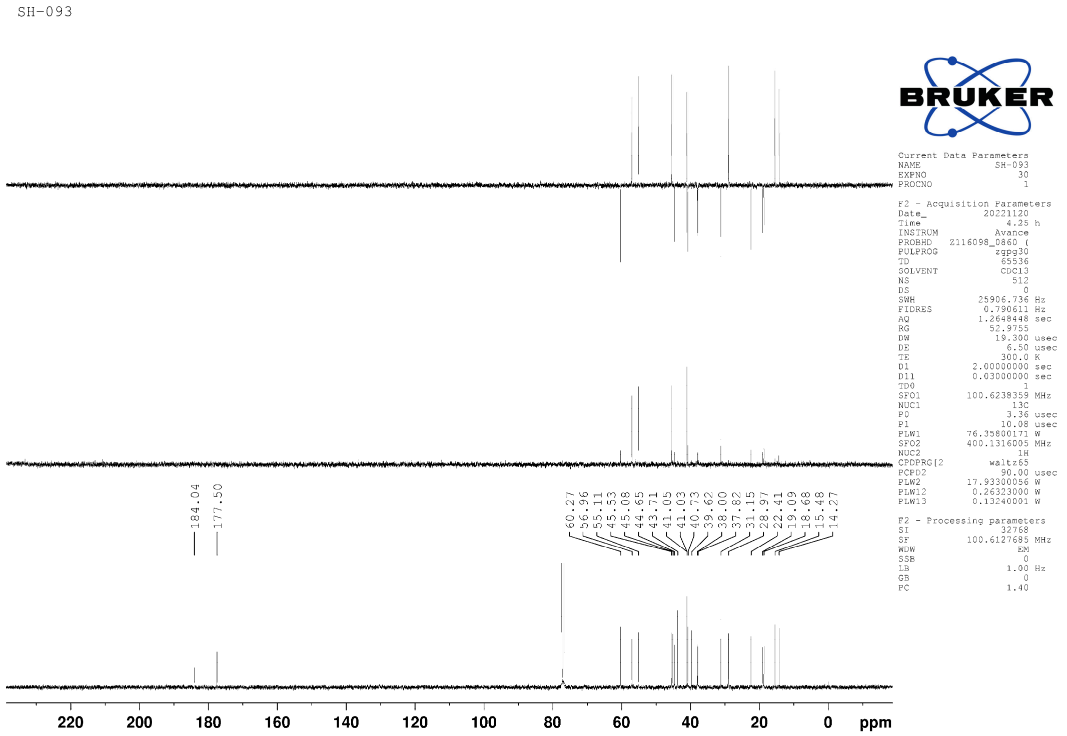

Figure S20. ^{13}C NMR (100 MHz) and DEPT spectra of sigesbeckin C (**3**) in $CDCl_3$.

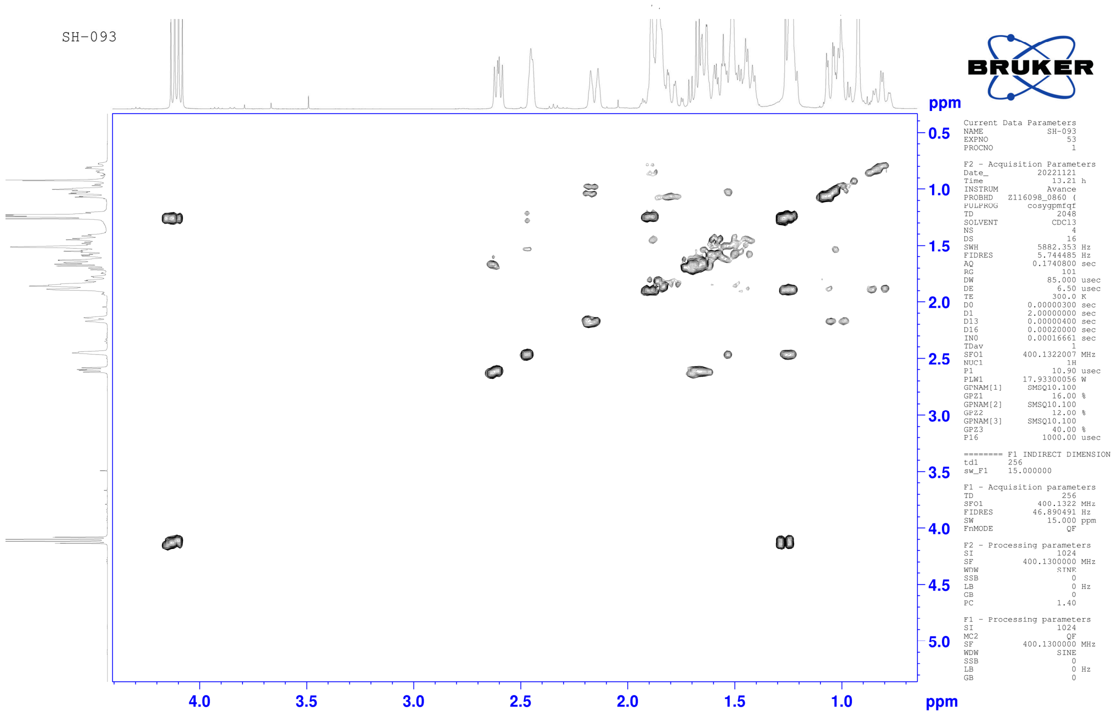

Figure S21. ^{1}H-^{1}H COSY spectrum of sigesbeckin C (**3**) in $CDCl_3$.

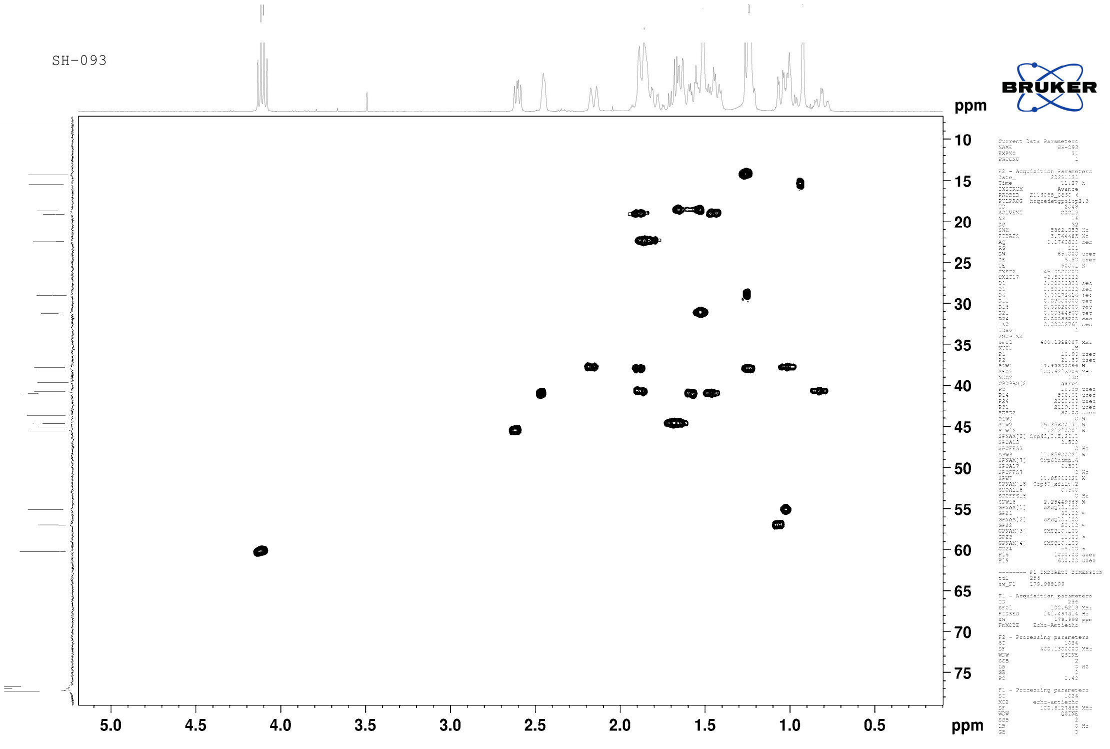

Figure S22. HSQC spectrum of sigesbeckin C (**3**) in $CDCl_3$.

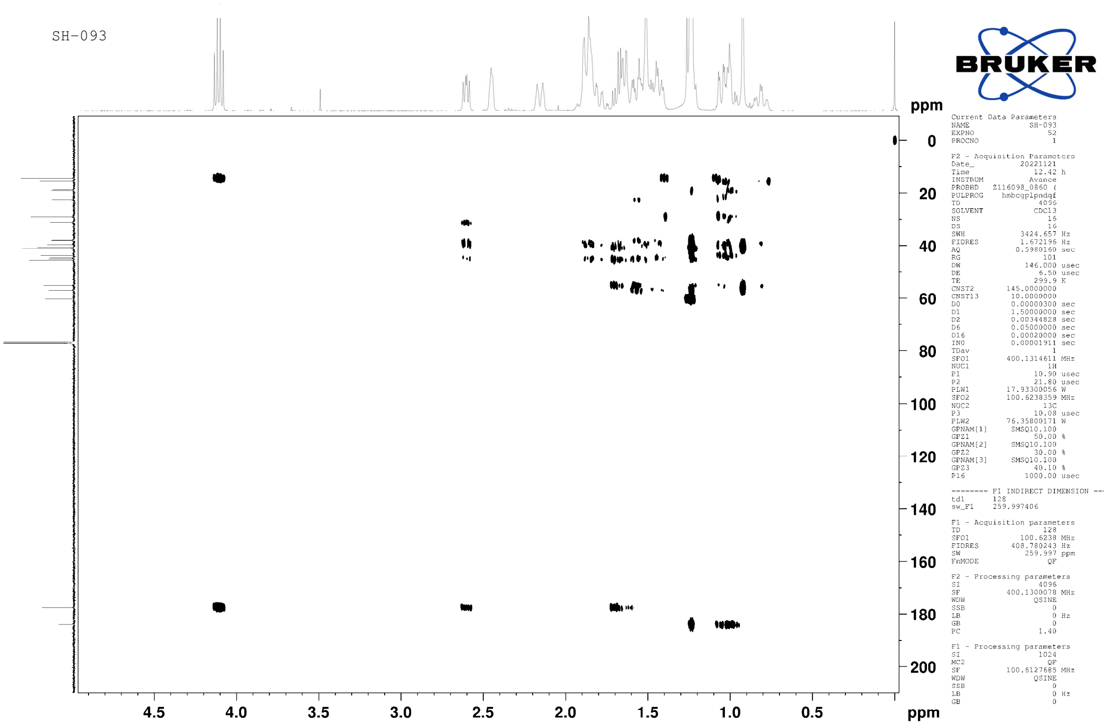

Figure S23. HMBC spectrum of sigesbeckin C (**3**) in CDCl3.

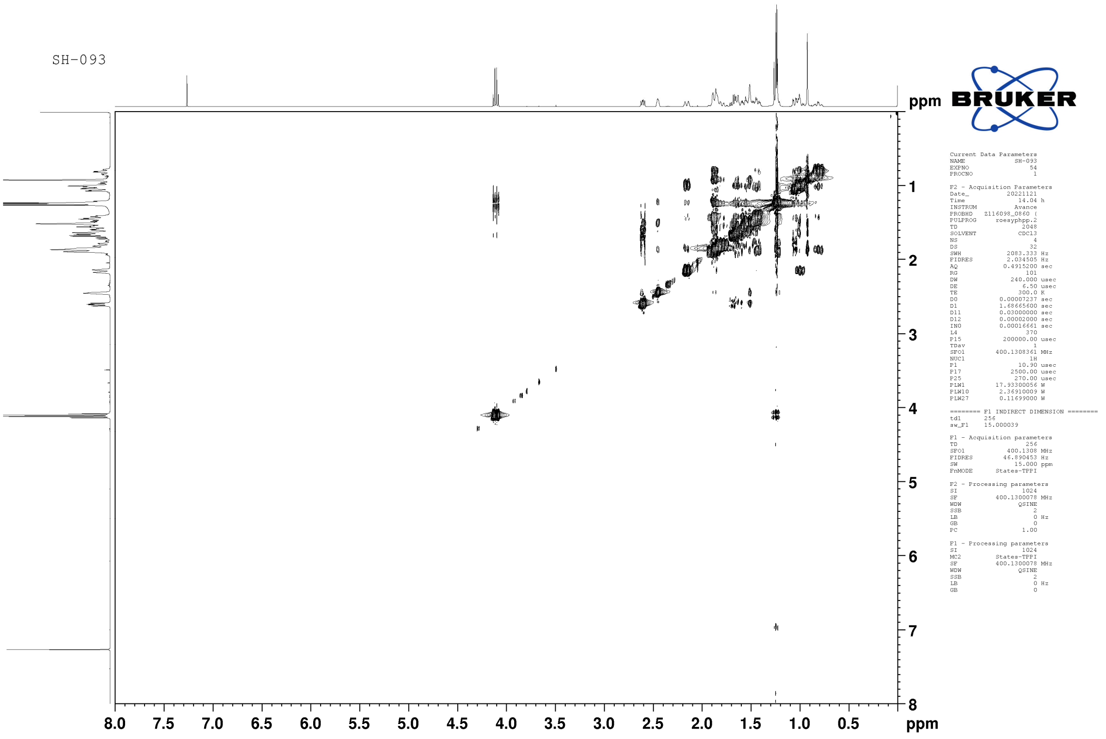

Figure S24. 1H-1H ROESY spectrum of sigesbeckin C (**3**) in CDCl3.

Qualitative Analysis Report

**Data File**	SH-093-N.d	**Sample Name**	SH-093-N
**Sample Type**	Sample	**Position**	P1-B5
**Instrument Name**	Instrument 1	**User Name**	
**Acq Method**	20220426-liangtong-N.m	**Acquired Time**	2022/11/14 12:06:21 (UTC+08:00)
**IRM Calibration Status**	Success	**DA Method**	Default.m
**Comment**			
**Sample Group**		**Info.**	
**Stream Name**	LC 1	**Acquisition Time (Local)**	2022/11/14 12:06:21 (UTC+08:00)
**Acquisition SW Version**	6200 series TOF/6500 series Q-TOF B.09.00 (B9044.0)	**QTOF Driver Version**	8.00.00
**QTOF Firmware Version**	25.723	**Tune Mass Range Max.**	3200

Spectra

Fragmentor Voltage	Collision Energy	Ionization Mode
120	0	ESI

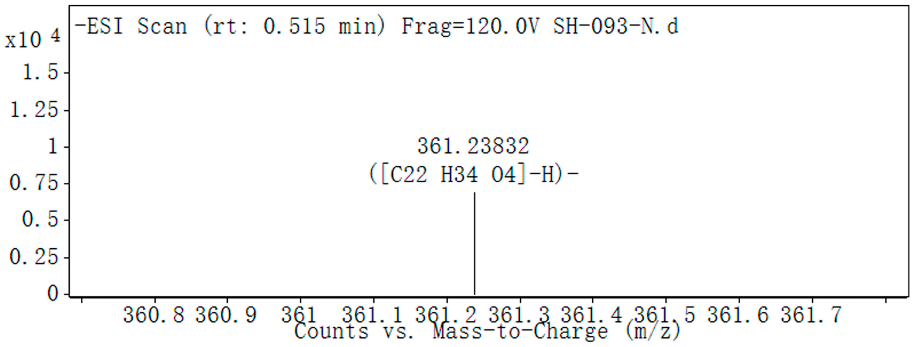

Spectrum Identification Results: - Scan (rt: 0.515 min) (SH-093-N.d)

Best	ID Sourc	Nam	Formula	Species	m/z	Scor	Diff (ppm	Score (MFG
☐	**MFG**		**C22 H34 O4**	**(M-H)-**	**361.2383**	**46.49**	**0.6**	**92.99**

Species	Lib/D	m/z	Score (iso. abund	RT	Score (mass	RT Dif	Score (MFG, MS/M	Score (MS	Score (MFG	Score (iso. spacing	Heigh	Ion Formula
(M-H)-		361.2383	80.56		99.71			92.99	92.99	94.45	6942.8	C22 H33 O4

Height (Calc)	Height Sum% (Cal	Height % (Calc	m/z (Calc	Diff (mDa	Heigh	Height	Height Sum	m/z	Diff (ppm
6650.8	78.1	100	361.23843	0.1	6942.8	100	81.6	361.2383	0.32
1617.9	19	24.3	362.24184	1.1	1317.3	19	15.5	362.2407	3.04
242.9	2.9	3.7	363.24467	-1.7	251.4	3.6	3	363.2463	-4.61

--- End Of Report ---

Figure S25. HRESIMS of sigesbeckin C (**3**).

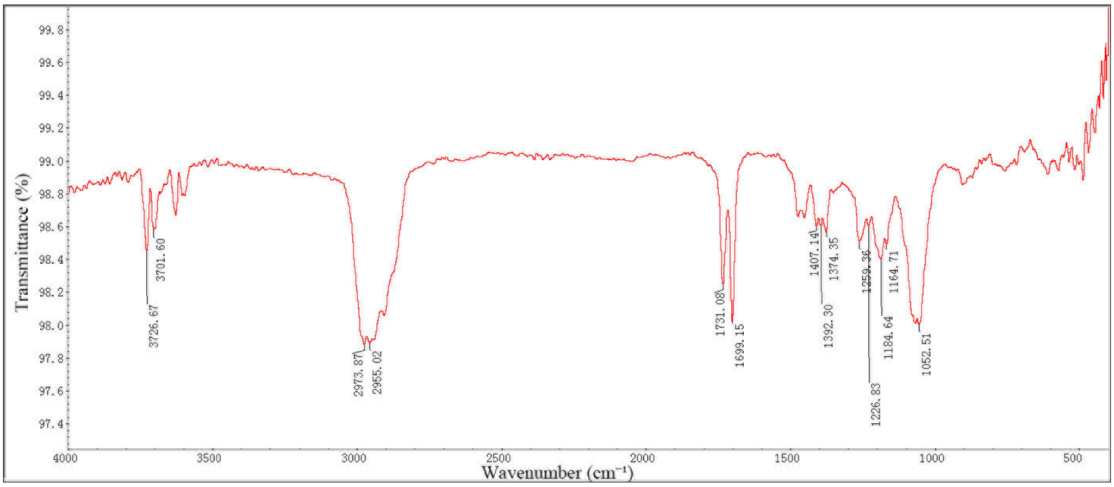

Figure S26. IR spectrum of sigesbeckin C (**3**).

Rudolph Research Analytical

This sample was measured by Autopol IV, Serial Number: 83650

Manufactured by Rudolph Research Analytical, Hackettstown, NJ, USA.

Measurement Date : 2024/8/7

Method Name : Specific Rotation @25C

Set Temperature : 25.0°C

Time Delay : 10

Delay between measurement : 1 Sec

N	Avg.	Std.Dev.	%RSD	Min	Max
5	-85.735	0.000	0.00	-85.735	-85.735

S.No	Sample ID	Time	Result	Scale	OR °Arc	WLG.nm	Lg.mm	Conc.g/100mL	Temp
1	SH-093-1	18:07:53	-85.735	SR	-0.1166	589	100	0.136	25.0°C
2	SH-093-1	18:08:00	-85.735	SR	-0.1166	589	100	0.136	25.0°C
3	SH-093-1	18:08:07	-85.735	SR	-0.1166	589	100	0.136	25.0°C
4	SH-093-1	18:08:14	-85.735	SR	-0.1166	589	100	0.136	25.0°C
5	SH-093-1	18:08:21	-85.735	SR	-0.1166	589	100	0.136	25.0°C

Page 1 of 1

Figure S27. Experimental ORD sigesbeckin C (**3**).

References

1. Zhu, Y.; Wang, Z.; Zhu, M.; Zhou, Z.; Hu, B.; Wei, M.; Zhao, Y.; Dai, Z.; Luo, X. A dual mechanism with H_2S inhibition and membrane damage of morusin from *Morus alba* Linn. against MDR-MRSA. *Bioorg. Med. Chem.* **2024**, *97*, 117544, doi:https://doi.org/10.1016/j.bmc.2023.117544.
